# Genomic analysis of head and neck cancer cases from two high incidence regions

**DOI:** 10.1371/journal.pone.0191701

**Published:** 2018-01-29

**Authors:** Sandra Perdomo, Devasena Anantharaman, Matthieu Foll, Behnoush Abedi-Ardekani, Geoffroy Durand, Luciana Albina Reis Rosa, Reetta Holmila, Florence Le Calvez-Kelm, Eloiza H. Tajara, Victor Wünsch-Filho, José Eduardo Levi, Marta Vilensky, Jerry Polesel, Ivana Holcatova, Lorenzo Simonato, Cristina Canova, Pagona Lagiou, James D. McKay, Paul Brennan

**Affiliations:** 1 International Agency for Research on Cancer (IARC), Lyon, France; 2 Institute of Nutrition, Genetics and Metabolism Research, Faculty of Medicine, Universidad El Bosque, Bogotá, Colombia; 3 Instituto de Medicina Tropical de SP Universidade de São Paulo- USP, São Paulo, Brazil; 4 School of Medicine of São José do Rio Preto, São José do Rio Preto, Brazil; 5 Faculdade de Saúde Pública, Universidade de São Paulo, São Paulo, Brazil; 6 Instituto Angel Roffo, Buenos Aires, Argentina; 7 Centro di Riferimento Oncologico (CRO), Aviano National Cancer Institute, Aviano, Italy; 8 Charles University of Prague, Prague, Czech Republic; 9 Laboratory of Public Health and Population Studies, Padova, Italy; 10 University of Athens Medical School, Athens, Greece; University of Cincinnati College of Medicine, UNITED STATES

## Abstract

We investigated how somatic changes in HNSCC interact with environmental and host risk factors and whether they influence the risk of HNSCC occurrence and outcome. 180-paired samples diagnosed as HNSCC in two high incidence regions of Europe and South America underwent targeted sequencing (14 genes) and evaluation of copy number alterations (SCNAs). *TP53*, *PIK3CA*, *NOTCH1*, *TP63* and *CDKN2A* were the most frequently mutated genes. Cases were characterized by a low copy number burden with recurrent focal amplification in 11q13.3 and deletion in 15q22. Cases with low SCNAs showed an improved overall survival. We found significant correlations with decreased overall survival between focal amplified regions 4p16, 10q22 and 22q11, and losses in 12p12, 15q14 and 15q22. The mutational landscape in our cases showed an association to both environmental exposures and clinical characteristics. We confirmed that somatic copy number alterations are an important predictor of HNSCC overall survival.

## Introduction

Head and neck squamous cell carcinomas (HNSCC) constitute a heterogeneous group of cancers, which include cancers arising at the oral cavity, nasopharynx, oropharynx, hypopharynx, and larynx. Collectively, these cancers are the seventh most common malignancy diagnosed worldwide [1], with areas of high incidence including Mediterranean Europe and South America [2]. Despite current therapeutic approaches, the prognosis is quite poor, with a 5-year survival ranging from approximately 25% to 60%, according to cancer subsite [3].

Cigarette smoking and alcohol abuse are the major risk factors, consistently associated with the incidence of head and neck cancers [4]. Additionally, human papillomavirus (HPV) infection is strongly associated with oropharyngeal cancer risk and prognosis, alongside a small number of other HNSCC [5]. Recent studies have highlighted the association between numerous differential genomic features and these exposures as well as clinical factors, providing insights for potentially improving prognostic risk stratification for HNSCC[6, 7]. The Cancer Genome Atlas TCGA has conducted the largest comprehensive genomic study of 528 HNSCC cases, consisting of an integrative analysis of multi-genomic data including somatic mutations, gene expression, methylation and miRNAs expression in a clinically and pathologically characterized dataset. The complete data analysis of a subset of 279 patients has allowed the description of the landscape of somatic genomic alterations and the identification of the principal molecular pathways involved in HNSCC development. Particularly, HNSCC are characterized by mutation of *TP53*, whole genome duplications and multiple recurrent chromosomal gains and losses associated to increased genomic disruption affecting cell cycle checkpoints and PI3K-AKT signaling[8, 9]. Increased rates of somatic copy number alterations (SCNAs) across the tumour genome are associated with poor prognosis and therefore it becomes important to identify SCNAs that might be functionally driving progression and outcome. In addition, genomic studies have revealed how differential genomic patterns among cases could identify various subgroups of tumours showing specific associations with histological subtypes, smoking, HPV status and overall survival[6, 10]

The principal objective of this study was to investigate whether somatic genetic changes identified in two large comprehensive case series in Europe and South America could influence the risk of HNSCC occurrence and outcome from those areas. A second objective was to investigate how somatic changes interact with environmental and host risk factors including HPV infection, alcohol and smoking. We selected 180 paired samples diagnosed as HNSCC from three multicentre studies representative of high incidence regions in Europe (ARCAGE study), Brazil (GENCAPO study) and Argentina (LA study); from which both tumour and blood samples were available in the IARC biorepository, along with complete epidemiological data.

## Materials and methods

### Study population and risk factor data collection

A total of 240 HNSCC cases were selected from three multicentre studies: two conducted in South America (LA study) between 1998 and 2002, and (GENCAPO study) between 1998 and 2008; and one completed in Europe (ARCAGE study) between 2002 and 2005. Selection of cases was based on availability for biological samples along with complete epidemiological and clinical data. However, no treatment information was obtained from most of these cases as this variable was not included in the original protocols. Extensive details of the three-large multicentre case-control studies are included elsewhere [11–13]. Briefly, all subjects underwent personal interviews to collect information on lifestyle exposures and hospital records were reviewed to obtain clinical and pathological information. All cases had biological samples collected at diagnosis and before any treatment [11–13]. Centralized HPV testing was completed for the three participating studies determined on serology testing as described before [14]. HPV positivity was defined based on HPV16 E6 status, which has been shown to be a highly sensitive and specific marker of HPV16-related oropharyngeal tumours [15–17]. Immunohistochemical evaluation of P16^INK4a^ expression and HPV DNA genotyping were also completed for a subset of samples using protocols previously described [12, 14], and these data were also used to confirm HPV status.

Informed consent was obtained from all participants in the three studies, and the analysis was approved by the Ethical Review Committee of the International Agency for Research on Cancer. All experiments were performed in accordance with relevant guidelines and regulations.

### Targeted sequencing

A customized gene panel of 14 genes (GeneRead DNAseq Custom Panels, Qiagen^®^) was used for targeted sequencing of tumour-blood pair cases. Gene selection was based on an independent analysis of TCGA data on HNSCC using MutsigCV algorithm complemented with the list of the most frequently mutated genes reported in the literature. Briefly, 20ng of DNA were used in multiplex PCR reactions using Qiagen^®^ recommended protocol. For library preparation, 100 ng of multiplex pools and the NEBNext End Repair Module (New England Biolabs, Ipswich, MA, USA) following manufacturer’s instructions. Individual barcodes (designed in-house and produced by Eurofins MWG Operon, Ebersberg, Germany) were ligated to each multiplex pool for sequencing. Both tumour and blood samples were sequenced at an average depth of 250X and 50X respectively using the PGM/PROTON^™^ Systems (Life Technologies, Carlsbad, CA, USA); sequences used for mutational calling had on target sequencing of 85%, and uniformity of 80–85%

### Mutational calling

Identification of somatic variants was performed using a recently developed statistical model called Needlestack[18] based on the idea that analysing several samples together can help estimate the distribution of sequencing errors to accurately identify variants. At each position and for each candidate variant, we model sequencing errors using a robust Negative-Binomial regression with a linear link and a zero intercept [19]. We calculate for each sample a p-value for being a variant (outlier from the regression) that we further transform into q-values to account for multiple testing. Needlestack has a detection limit of variant allelic fractions between 0.05% and 0.5% depending on the error rate at the base change considered (ranging from 0.001% to >10% at homopolymers) and the sequencing depth. Needlestack is free and open-source and is available publicly as a beta version under https://github.com/IARCbioinfo/needlestack. A detailed description of the *Needlestack* variant caller has been previously published [20, 21]. Variant calls were annotated using ANNOVAR [22] and indels, nonsense, splicing, or missense variants were only kept for subsequent analyses if reported in COSMIC-76 and/or classified as deleterious, disease causing or damaging in at least one of the five variant classification databases (SIFT, Polyphen, MutationTaster, MutationAssessor, FATHMM, LR) (S3 Table).

Filtering of VCF calls was done using a threshold of 0.5% allelic fraction, minimal read depth of 100X and minimal phred-scaled q-value of 30. Removal of germline variants was additionally confirmed by comparison of corresponding paired blood sequences; all filtered variants were manually curated by inspection of BAM files using the Integrative Genomics Viewer (IGV) 2.3 (Broad Institute, Cambridge, MA, USA).

Internal technical validation of both the sequencing procedure and the mutational calling was done by including 10% of samples as technical replicates in each library preparation. Additionally, an independent library preparation including a random selection of 20 tumour samples was sequenced and analysed independently and results were 100% concordant. All cases from the GENCAPO study had been previously sequenced for *TP53* mutations using Sanger sequencing, which we used to further validate our mutational calls and compare them with a different calling method (GeneRead Panel Variant Calling analysis tool from Qiagen^®^) (S1 Fig).

### Somatic copy number alterations (SCNAs)

DNA from each tumour was hybridized to Illumina HumanCytoSNP-12v2.1 arrays using standard manufacturer’s protocol. Formalin-fixed paraffin-embedded (FFPE) samples underwent a quality control assay using the Illumina FFPE QC Kit, samples were selected based on a ΔCq below or equal to 2 and then restored using the Infinium HD FFPE Restore Protocol. We included 10% of technical and biological replicates for quality control and validation. Microarray data are available in the ArrayExpress database (www.ebi.ac.uk/arrayexpress) under accession number E-MTAB-4863. The R package crlmm [23] was used for pre-processing, genotyping and calculation of circular binary segmentation to estimate the normalized copy number. Germline copy number alterations were removed using the Database of Genomic Variants [24]. Identification of significant amplified or deleted regions was performed by using GISTIC 2.0 [25] using 99% confidence level and q-value threshold 0.25. Focal amplification or deletion for all the 14 genes sequenced was determined only using the GISTIC copy number value 2 or -2 respectively as the true value. OncoPrinter and MutationMapper tools were used for visualization of mutational data [26, 27]. Integrative cluster analysis of mutation and copy number data was performed using the R package iClusterPlus [28].

### Statistical analysis

Mutual exclusion and co-occurrence test for mutations (including both single nucleotide variants and copy number alterations) found in the 14 genes evaluated, were based on weighted permutations assessing the deviation of the observed coverage compared to expected obtained by permuting events [29]. Fisher exact test was used to determine the relationship of clinical characteristics in the 3 studies. For each patient, time at risk was calculated from cancer diagnosis to death or end of follow up (Last Follow up date: 30/01/2013 for the ARCAGE study, 30/06/2009 for GENCAPO and 30/06/2006 for the LA study). Follow-up was censored at 5 years, given that most cancer related events occur before that time. The Kaplan-Meier estimator was used to estimate the distribution of the 5-year survival. Multivariate Cox proportional hazard models were used to estimate HRs and their corresponding p values for all candidate risk factors and genomic biomarkers. Age, subsite, stage, nodal status (defined by pathological nodal stage), smoking and alcohol status were used as covariates. A correction for multiple-hypothesis testing was employed using the method of Benjamini and Hochberg [30] Log-rank test was used to compare the different survival distributions.

## Results

### Epidemiological description of the three studies

**Fig 1:**
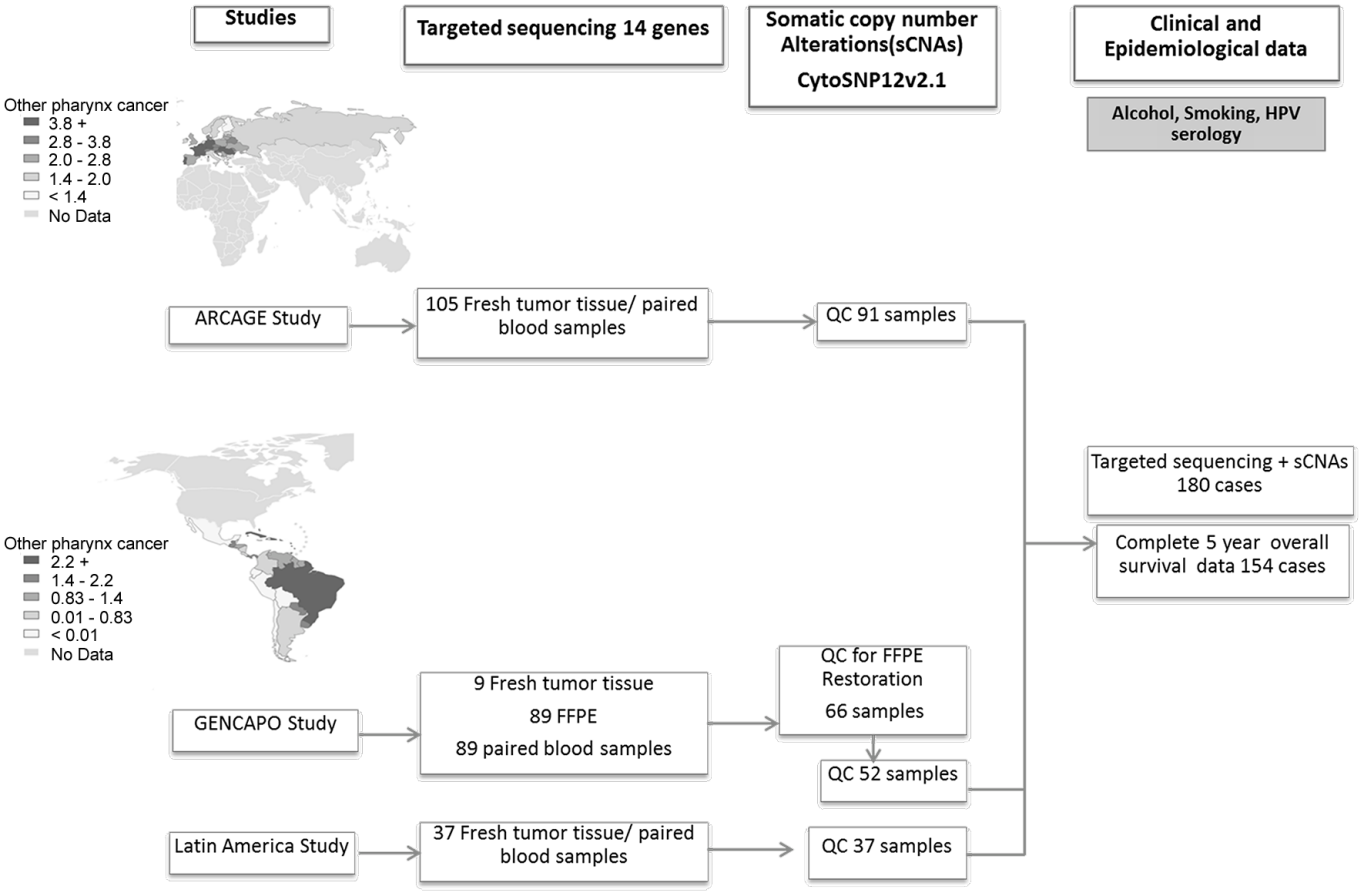
Workflow of processing and analysis of HNSCC samples from the three different studies. QC for copy number evaluation: Quality control of samples based on signal to noise ratio>5.0. Maps show estimated age-standardized incidence rates for HNSCC (other pharynx sites) in Europe and South America. [31].

A total of 180 cases had complete sequencing and copy number information (Fig 1). Clinical and pathologic characteristics of cases in the three studies are described in Table 1. Consistent with previous reports the majority of the cases were males (82%), current smokers (67%) and current drinkers (70%). Mean age at diagnosis was 59 years (range 18–88 years). Thirty-three- percent of all cases were diagnosed with oral cavity cancer, 25% with oropharyngeal cancer, 18% with laryngeal cancer, 7% with hypopharyngeal cancer and 16% with overlapping topographies. Seventy-percent of all cases presented advanced disease (stages III-IV). The majority of non-smokers (80%) and oropharyngeal cases (67%) were part of the European study (ARCAGE). Fifteen cases out of 180 (8%) were classified as HPV16 positive, 73% of which were oropharyngeal cases.

**Table 1:** Clinical and epidemiological description of 180 HNSCC cases from the three studies.

	STUDY							
	ARCAGE (Czech Republic, Italy, Greece)		GENCAPO (Brazil)		LA (Argentina)		Total	
Sex*	n	**%**	n	**%**	n	**%**	n	**%**
Female	26	**28.57**	2	**3.85**	5	**13.51**	33	**18.33**
Male	65	**71.43**	50	**96.15**	32	**86.49**	147	**81.67**
**Age group**								
18 to 50	18	**19.78**	9	**17.31**	7	**18.92**	34	**18.89**
51 to 60	25	**27.47**	28	**53.85**	14	**37.84**	67	**37.22**
61 to 70	28	**30.77**	10	**19.23**	9	**24.32**	47	**26.11**
>70	20	**21.98**	5	**9.62**	7	**18.92**	32	**17.78**
Subsite*								
Oral cavity	32	**35.16**	14	**26.92**	13	**35.14**	59	**32.78**
Oropharynx	30	**32.97**	10	**19.23**	5	**13.51**	45	**25**
Hypopharynx	2	**2.2**	11	**21.15**	0	**0**	13	**7.22**
Larynx	17	**18.68**	3	**5.77**	12	**32.43**	32	**17.78**
Overlapping	10	**10.99**	12	**23.08**	7	**18.92**	29	**16.11**
No information	0	**0**	2	**3.85**	0	**0**	2	**1.11**
Stage*								
T1	8	**8.79**	1	**1.92**	0	**0**	9	**5**
T2	25	**27.47**	9	**17.31**	0	**0**	34	**18.89**
T3	16	**17.58**	17	**32.69**	3	**8.11**	36	**20**
T4	40	**43.96**	19	**36.54**	34	**91.89**	93	**51.67**
No information	2	**2.2**	6	**11.54**	0	**0**	8	**4.44**
Nodal Status*								
N0	50	**54.95**	14	**26.92**	3	**8.11**	67	**37.22**
N+	38	**41.76**	27	**51.92**	8	**21.62**	73	**40.56**
No information	3	**3.30**	11	**21.11**	26	**70.27**	40	**22.22**
Smoking*								
Non-smoker	18	**19.78**	1	**1.92**	3	**8.11**	22	**12.22**
Former smoker	13	**14.29**	14	**26.92**	6	**16.22**	33	**18.33**
Current smoker	60	**65.93**	32	**61.54**	28	**75.68**	120	**66.67**
No information	0	**0**	5	**9.62**	0	**0**	5	**2.78**
Alcohol*								
Non-drinker	7	**7.69**	2	**3.85**	6	**16.22**	15	**8.33**
Former drinker	11	**12.09**	18	**34.62**	4	**10.81**	33	**18.33**
Current drinker	73	**80.22**	26	**50**	27	**72.97**	126	**70**
No information	0	**0**	6	**11.54**	0	**0**	6	**3.33**
**HPV Status (HPV16E6 serology)**								
Negative	82	**90.11**	47	**90.38**	34	**91.89**	163	**91.57**
Positive	9	**9.89**	3	**5.77**	3	**8.11**	15	**8.43**
No information	0	**0**	2	**3.85**	0	**0**	2	**1.11**

*p value<0.05

### Mutational profile of the 14 gene panel in cases

**Fig 2:**
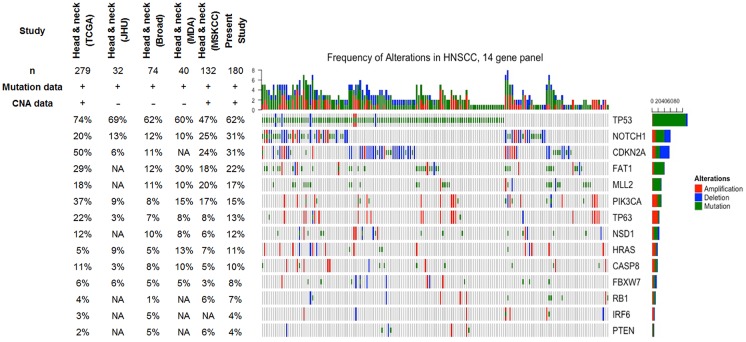
OncoPrint diagram of mutational frequencies and types of alterations of the 14 genes sequenced. Only altered samples are shown. Rows are sorted based on the frequency of the alterations in all samples and columns are sorted to visualize the mutual exclusivity across genes. Frequency of mutations for the following Head and Neck cancer publications are shown: Head & Neck (TCGA)[10], Head & Neck (JHU)[39], Head & Neck (Broad)[32], Head & Neck (MDA)[40], Head & Neck (MSKCC)[41]. NA: Not available.

Ninety four-percent of all sequenced cases had at least one alteration (single nucleotide variants (SNVs) or amplification/deletion) in any of the 14 genes selected (Fig 2) (S3 Table). The overall frequency of alterations for the 14 genes was similar to previous publications with a higher enrichment of alterations in the *TP53*, *NOTCH1* and *CDKN2A* genes [10, 32–34]. Among the 10 cases without alteration in the 14 genes, 4 corresponded to HPV positive cases (S4 Fig).

*TP53*, *FAT1*, *MLL2* and *NOTCH1* were the genes more frequently altered by single nucleotide variants (SNVs) (Fig 2). As previously described [35, 36], *TP53* mutation was mostly prevalent in HPV negative tumours (only three out of 15 HPV16 positive tumours harboured a *TP53* mutation, and all three cases were current smokers) (S4 Fig). *TP53* mutations clustered predominantly in DNA binding domains, particularly in hotspot codons 175, 248, 249, 273 and 282 (S3 Fig). Forty-four-percent of all mutations were classified as disruptive mutations according to the definition by Poeta and colleagues[37]. Fifty-five-percent of all *TP53* SNVs were missense mutations and from those 64% were classified as high-risk mutations based on the evolutionary action score EAP53[38]. *FAT1*, *MLL2* and *NOTCH1* mutations (missense and truncating mutations) were distributed along the gene coding region and did not show mutational enrichment of specific protein domains (S3 Fig).

Mutual exclusive alterations were identified between genes with recognized activity in the same signalling pathway, suggesting overlapping functional consequences of those mutations. This included *TP53* and *PIK3CA* (p<0.001), both involved in cell cycle control and survival, and *NOTCH1* and *TP63* (p = 0.003) genes, which play important functions of squamous cell differentiation (S2 Fig).

Significant co-occurring alterations were found principally in the *TP63* and *PIK3CA* genes (p<0.001), both genes located on a frequently amplified region (3q) along with concomitant alterations in *HRAS* and *NOTCH1* genes (p<0.001).

### Somatic copy number alterations (SCNAs)

**Fig 3:**
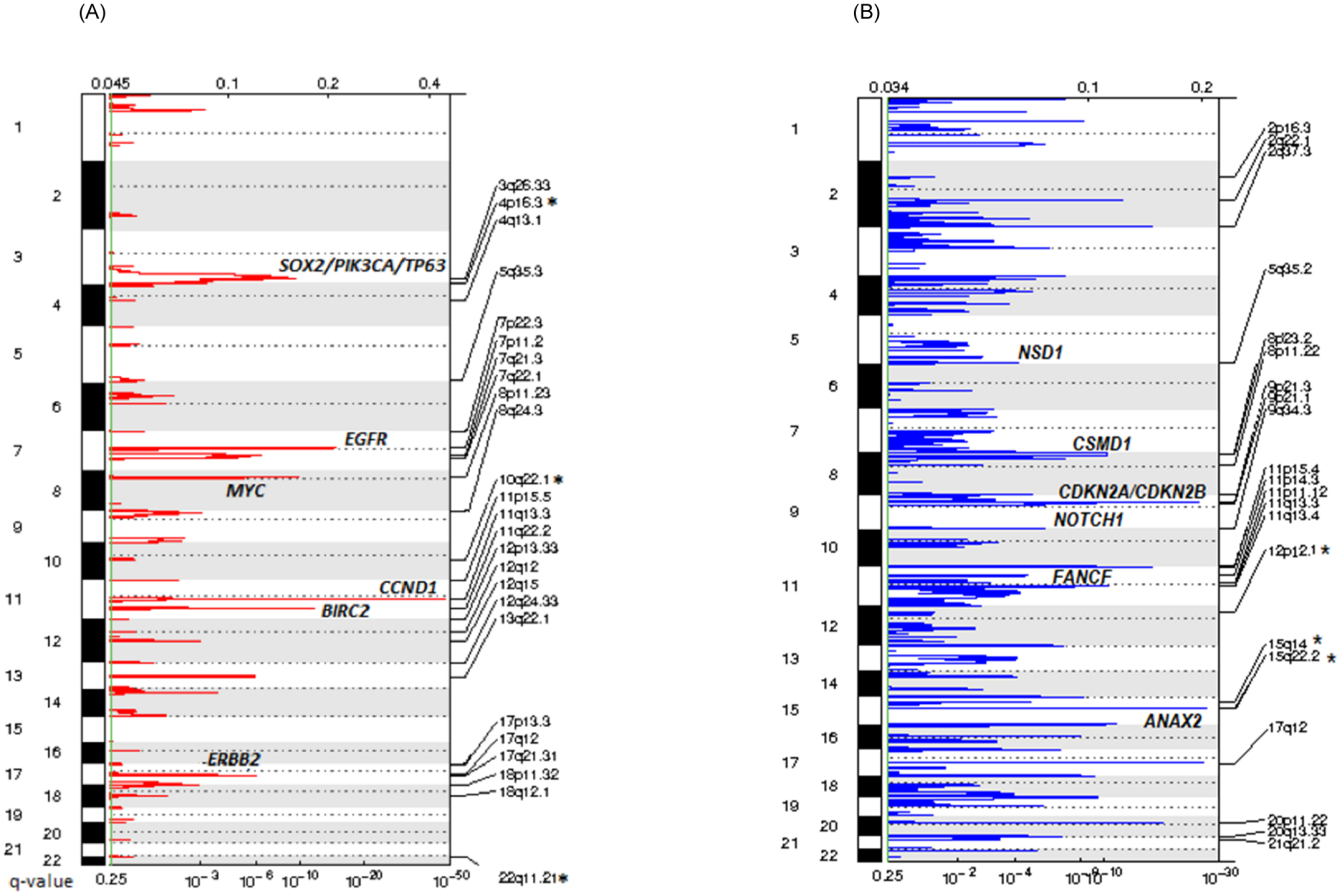
Diagram of significant focal copy number alterations. FDR (Top) and q-values of the alterations are shown in each panel. **(A)** Copy number gains **(B)** Copy number losses. Selected associated genes in some regions are shown. (*) Regions significantly associated with overall survival.

Overall, cases were characterized by low chromosomal instability represented by a low copy number burden (mean 23 alterations included amplifications and deletions) compared to the TCGA dataset [10]. We found a total of 47 significantly recurrent amplified regions and 69 deleted regions (q-value<0.1) (Fig 3 and S1 and S3 Tables). The most recurrent focal amplified region was 11q13.3 including the *CCND1* and *FGF3* genes amplified in 40% of samples (60/66 with smoking history), consistent with a region preferentially amplified on smoking related tumours [10, 42]. In addition, we identified regions harbouring oncogenes frequently activated in HNSCC as previously described [10, 32, 33, 39, 40]: 11q22 (*BIRC2*), 3q26 (*SOX2*, *PIK3CA*), 3q28 (*TP63*), 7p11 (*EGFR*), 17q12 (*ERBB2*), along with amplification of regions 8p11, 13q22 and 7q22.

The most frequently deleted region was 15q22, including the locus of the *ANXA2* gene that has been previously found to be downregulated in both head and neck dysplasia and HNSCC [43, 44]. Additionally, recurrent focal deletions were present in cases, particularly at three regions on chromosome 11 (11p15-p15.5, 11q13-q13.3 and 11q23-q24) previously identified as being of frequent microsatellite instability and/or loss of heterozygosity (MSI/LOH) in HNSCC. We also identified deletions in regions of commonly described transcription regulators and tumour suppressor genes in HNSCCs [10, 45]: 5q35.2 (*NSD1*), 20p11 (*NKX2-2*), 8p22.2 (*CSMD1*), 9q34.3 (*NOTCH1*); together with loss of 9p21.3 containing the *CDKN2A* gene which was found almost exclusively in HPV negative tumours (deletion in 1 out of 15 HPV positive cases) (S4 Fig).

Comparison of copy number alterations based on HPV16 status showed a lower proportion of significantly altered regions in HPV positive cases. In particular, the 11q24.3 region (containing the *ATM* and *APLP2* genes) was differentially lost in HPV positive cases (S4 Fig). Additional losses in the 6p region, close to the HLA class I genes loci, were also identified in HPV positive cases.

### Integrated analysis

**Fig 4:**
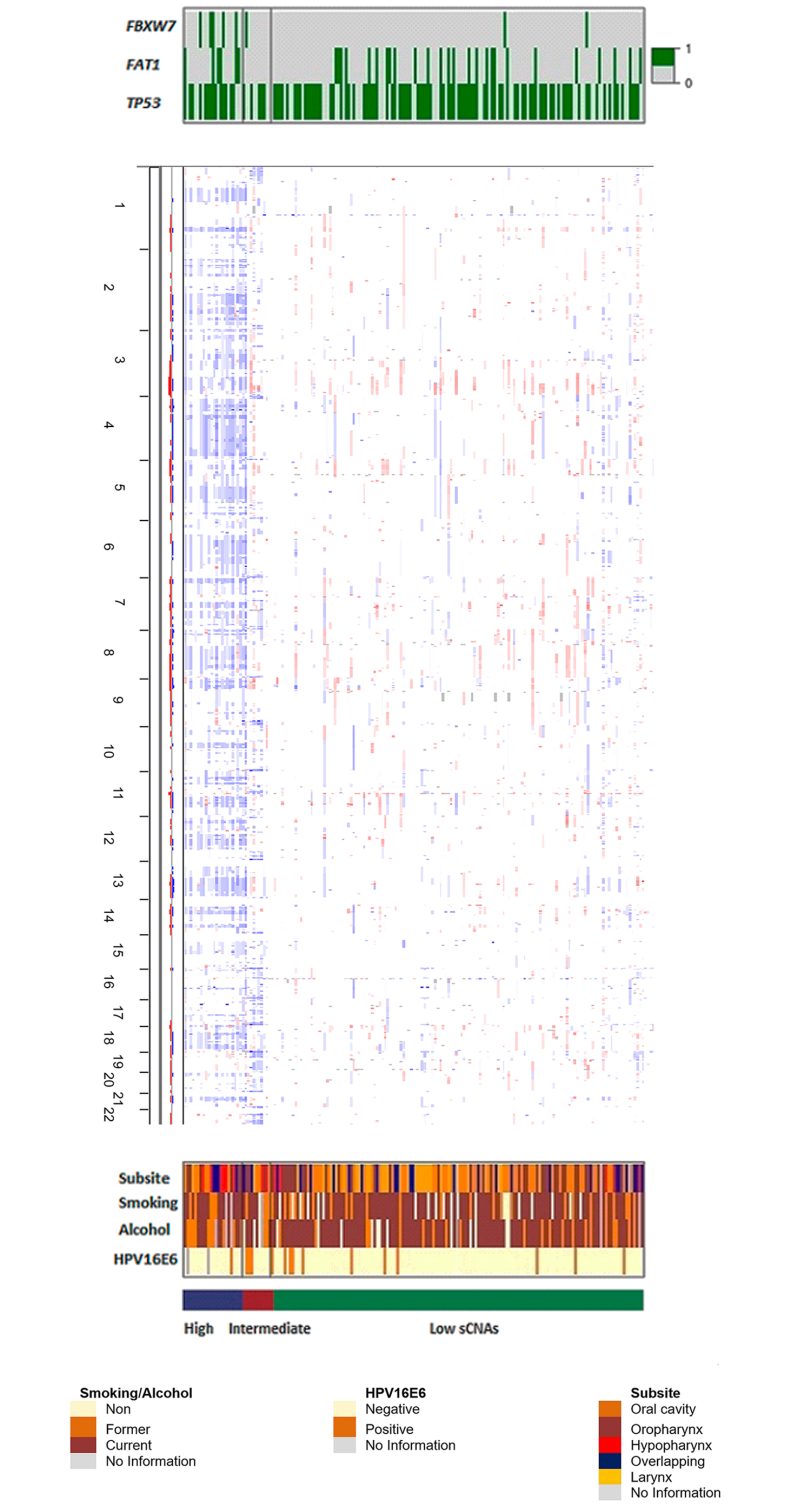
Integrative cluster analysis plot. Cases are grouped by mutation and SCNA status. Top panel: only significant clustering genes are shown (0 = non-mutated, 1 = mutated), middle panel: SCNAs. Amplified (red) and deleted (blue) chromosomal regions. Altered regions are arranged vertically and sorted by genomic locus, with chromosome 1 at the top of the panel and chromosome 22 at the bottom, lower panel: colour coded clinical and epidemiological characteristics.

Integrative cluster analysis of both mutational and copy number data identified three distinct clusters with major genomic features including *TP53*, *FAT1* and *FBXW7* SNVs and low, intermediate and high genomic instability. The *FBXW7* gene was significantly mutated in both groups with high and intermediate SCNAs (Fig 4). Eighty-percent of total cases were clustered in the low SCNAs group (mean copy number events = 19). The intermediate SCNAs group (mean copy number events = 39) had only advanced cases (11) and the high SCNAs group (mean copy number events = 43) clustered only cases from Brazil with history of alcohol and smoking exposure (23 cases).

### Survival analysis

**Fig 5:**
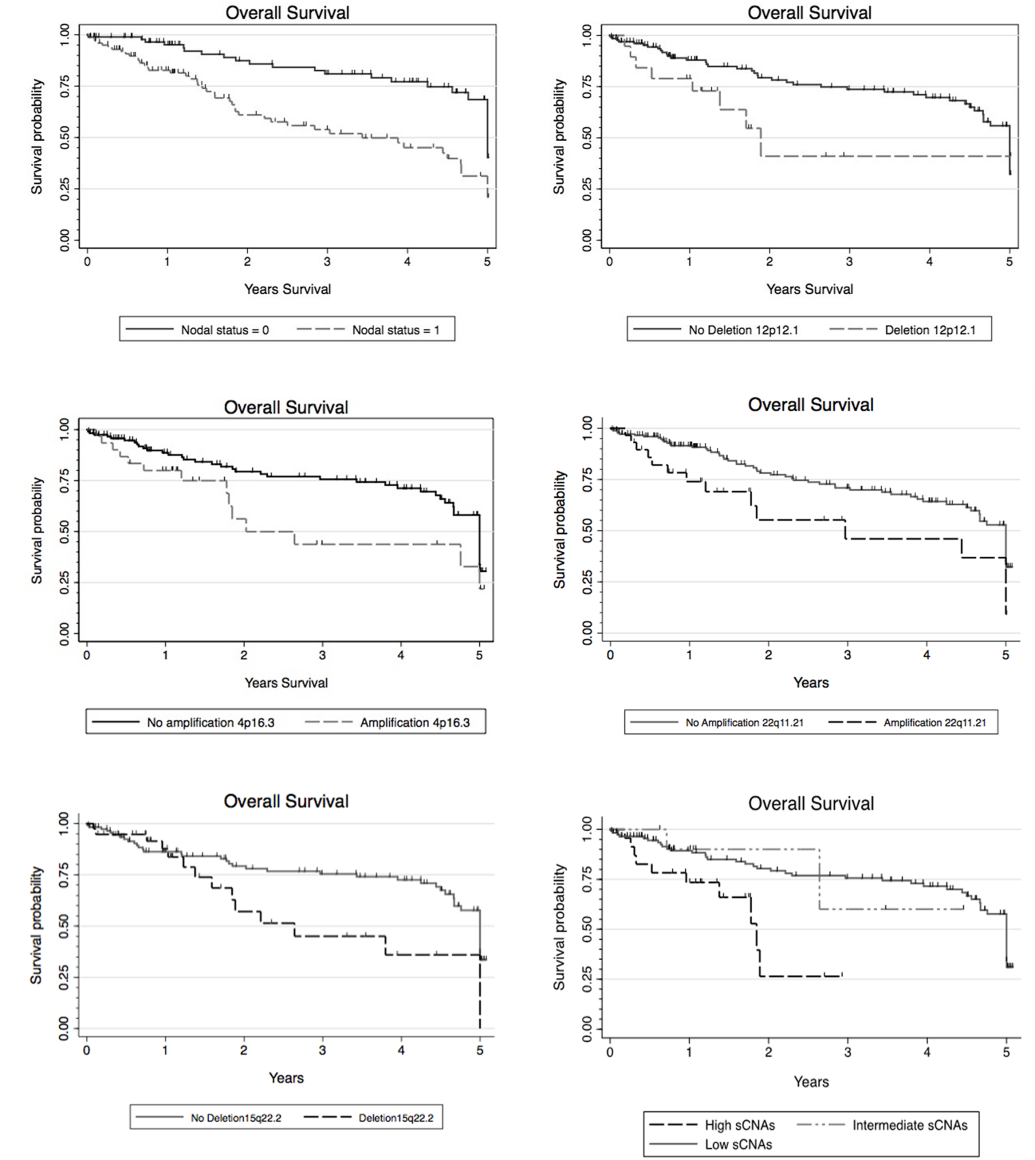
Kaplan-Meier curves showing overall survival outcome for nodal status, significant focal copy number alterations in 22q11.2,15q22 and 12p12 regions associated to smoking and advanced stage, amplification in 4p16.3 and for the three SCNAs clusters.

Survival data was available for 154 cases (Fig 1). Age and nodal status were the only clinical or demographic variables significantly associated to overall survival (p = 0.01) (Fig 5 and S2 Table). Multivariate analysis including each of the 14 genes sequenced showed no association with overall survival. Further analysis of *TP53* mutational status showed no association between mutation type (either disruptive/non-disruptive or EAP53 score of missense mutations) and overall survival (S2 Table).

Analysis of the most frequently focal SCNAs showed significant associations between the amplified regions 4p16, 10q22 and 22q11 and a reduction in overall survival. We found additional associations between losses in regions 12p12, 15q14 and15q22 and decreased overall survival (Fig 5 and S2 Table). Although individual candidate genes in these regions were difficult to identify due to the large number of enclosed genes (>20), we identify some genes that have been previously altered in HNSCC (S2 Table) and have been included in our discussion below.

Our integrative clustering approach based on copy number events was also associated with improved overall survival for cases clustered in the low copy number group (p = 0.01) (Fig 5 and S2 Table).

## Discussion

Head and neck carcinomas show common genomic features determined by SNVs and copy number events in driver genes and cellular pathways associated to the common histology of squamous cell types. However, there is broader genomic heterogeneity due to the variability in anatomic subsite location and the interaction of multiple risk factors such as alcohol and tobacco exposure as well as HPV infection.

Even though we limited our sequencing study to 14 genes, our results showed that most of the mutations described in these genes are representative of the mutational profile of head and neck cancer cases (mutations in 94% of cases). Additionally, the mutational frequency in all 14 genes was comparable to the frequencies observed in previous publications from the largest sequencing projects of Head and Neck cancer cases. In future studies, inclusion of some additional genes such as *AJUBA*, *HLA-A/B*, *NFE2L2*, *KRAS*, *FGFR2/3* and *TRAF3* could improve mutation detection and better capture the mutational landscape of HPV positive tumours, as well as favour the understanding of additional cellular and molecular mechanisms involved in tumour development such as the oxidative stress pathway.

The predominance of low SCNAs in our cases confirms previous studies that differentiate subsets of head and neck tumours (described as M-mutational class tumours) characterized predominantly by mutations rather than chromosomal instability events [8]. A subclass of these low SCNAs group is enriched with alterations in the *PIK3CA-AKT* and p53-mediated apoptosis pathways, in agreement with the number of alterations in *TP53*, *CDKN2A* and *PIK3CA* we observed in our cases.

Eight percent of all cases were HPV 16 positive and 73% corresponded to oropharyngeal tumours. The reduced number of oropharyngeal tumours in the study (25%) and the predominance of older cases, current smokers and drinkers, characteristics preferentially associated to non-related HPV HNSCC[46], might account for the low number of HPV positive cases. In addition, half of our study cases were from Brazil and Argentina which could contribute to the low percentage of HPV positive HNSCC, as it has been previously described in South America [12, 47]. Despite of the limited number of HPV positive cases in our series, we established that HPV positive tumours remain a distinct subset characterized by lower somatic copy number events and differential mutation patterns [36, 48, 49]. Loss of the 11q24.3 region which contains both *ATM* and *APLP2* genes, is a frequent alteration in HPV positive cases[48]. Moreover, the *APLP2* gene is related to tumour immunology as it regulates surface expression of the MHC class I molecules[50, 51]. These results suggest that alterations related to immunological responses might differentiate infection related HNSCC tumours. Further characterization should however, be performed for this group particularly to address the associations between genomic alterations and smoking and alcohol exposure and a differentiated analysis by histological subsite.

Our results confirmed that somatic copy number alterations are an important predictor of overall survival. We have described an improved overall survival for those cases with low SCNAs. These results are in agreement with recent observations showing the direct association between low copy number events, intratumour heterogeneity and clonality with genomic instability and how the joint effect of these factors might influence survival [10, 52–54]. Recently, Andor and colleagues analysed clonality across 12 cancer types from the TCGA dataset, including head and neck cancer cases, and showed that intratumour heterogeneity levels above or below an intermediate measure of clonality were associated with significantly reduced risk of mortality. Moreover, they used copy number alteration abundance as a surrogate measure of genomic instability and found that when SCNAs were present either in a low or a high fraction of the tumours, cases had an improved survival [53]. The high SCNAs group in our study showed the lowest overall survival and clustered only samples from Brazil, all characterized by higher stage and history of both smoking and alcohol exposure. These results give additional evidence to support the rise in mortality due to this malignancy in this country [55].

The mutational profile described in our series of cases showed a clear association to both environmental exposures and clinical characteristics including associations with overall survival. We found that both mutational and focal copy number alterations were correlated with genetic alterations previously described for smoking related head and neck cancers as well as for biomarkers of late stage tumours [10, 32]. Alterations exclusively found in cases with history of both smoking and alcohol consumption included 5q35.3 amplification and 11p14.3 deletion. This last region is of interest as it encloses the *FANCF* gene, involved in the Fanconi anemia pathway and commonly associated to squamous cell carcinoma susceptibility. In addition, *FANCF* inactivation has been previously related to chromosomal instability on sporadic HNSCC [56].

Additionally, focal copy number alterations were found to be significant prognostic markers: 22q11.2 amplification and deletions in 15q22 and 12p12 regions have been associated to smoking related tumours and advanced stage. The 22q11 region contains the *CRKL* gene, which has been characterized as an oncogene in lung SCC [57] and as a promoter of cell growth, motility and adhesion during HNSCC tumorigenesis [58]. Decreased survival in cases with loss of 12p12.1 region, locus of the *PIK3C2G* gene, showed a HR of 3.0 95% CI [1.2; 7.77]. Advanced stage HNSCC tumours have shown mutations in more than one PI3K pathway molecule: *PIK3CA*, *PTEN* and described alterations in *PI3C2G* [59, 60]. Moreover, the 15q22 region, locus of the *ANXA2* gene, has been previously shown to be associated with poorly differentiated tumours in advanced cases. Decreased *ANXA* expression has not however, been formerly shown to be an independent prognostic factor for disease-specific survival in HNSCC [43, 44]. We report for the first time an association between decreased overall survival and amplification of the region 4p16.3, locus of the *FGFR3* gene. High expression levels of FGFR3 contribute of tumour initiation and early-stage progression in HNSCC[61]. More importantly, preclinical studies have demonstrated that FGFR inhibition reduced cell proliferation and increased cell apoptosis in head and neck cancer *in vitro* and *in vivo*[62], highlighting the potential prognostic and therapeutic role of *FGFR3* in HNSCC.

Most studies on HNSCC have documented a decreased overall survival associated to *TP53* mutations[6, 37, 63]. Our study, however, did not find any association between the mutational status of the 14 genes sequenced and overall survival. A specific analysis based on *TP53* mutation type (disruptive vrs nondisruptive or EAP53 score of missense mutations) showed no association to overall survival, either. In agreement to our results, Kim and colleagues, found that patients diagnosed with oral squamous cell carcinoma of the gingivo-buccal region (GBSCC) from the Indian Team project of the International Cancer Genome Consortium (ICGC), did not showed an association between *TP53* mutation status and overall survival [64]. Similar to the epidemiological and clinical characteristics of our study cases, GBSCC patients from the ICGC study were most exposed to tobacco and/or alcohol, presented advanced stage (III/IV) and half of the cases had confirmed nodal metastasis [33].

One of the main limitations of our study is the reduced number of HNSCC cases with early stage tumours. It would be important to further characterize the genomic alterations in early stages of head and neck cancer cases in order to identify biomarkers for early detection and prognostic stratification especially for the high-risk groups in regions of increase incidence. In addition, our survival analysis was limited due to the lack of complete treatment information for most cases. Treatment regimens have an important association with Head and Neck cancer overall survival and should be included in future analysis specially those involving multicentre studies[65].

In summary, we have identified HNSCC cases with low SCNAs that differentiate as a subset of head and neck cancers driven predominantly by gene mutations and focal alterations rather than chromosomal instability events and are characterized by an improved overall survival. The mutational landscape described in our series of cases showed a clear association to both environmental exposures (alcohol and smoking consumption and HPV infection) and clinical characteristics. Further studies integrating genomic, clinical and epidemiological data, especially in high-risk populations, are necessary to better identify high-risk stratification and characterize prognosis of head and neck cancer cases.

## Supporting information

S1 Fig Mutation calling validation. (A) Venn diagram of number of *TP53* mutations detected in the Gencapo Series. Example: *TP53*Asn239Asp mutation previously detected by Sanger sequencing (B) Plots of mutational calling showing an example of independent libraries sequenced from the same case.
(PDF)

S2 Fig (A) Mutually exclusive alterations between the 14 genes sequenced (Significance p-value of mutual exclusivity derived from the Z-score) (B) Co-occurrence of alterations (Significance p-value of co-occurrence derived from the Z-Score). Z score based on deviation of the observed mutations compared to expected, obtained by permuting events.
(PDF)

S3 Fig Diagrams of mutation distribution in genes with frequent SNVs. Mutation colours represent: Green: Missense Mutations; red: Truncating Mutations (Nonsense, Nonstop, Frameshift deletion, Frameshift insertion, Splice site), black: Inframe Mutations (Inframe deletion, Inframe insertion). Circles colored with purple indicate residues that are affected by different mutation types at the same proportion.
(PDF)

S4 Fig Mutational Profile and copy number losses in HPV positive cases. (A) Mutational frequencies of the 14 genes sequenced in 15 HPV16E6 positive cases. (B) Comparison of Significant Focal copy number losses between HPV positive and HPV negative cases. (*) Regions significantly associated with overall survival.
(PDF)

S1 Table GISTIC list of focal copy number amplifications and deletions. In red regions significantly associated with overall survival and head and neck cancer related genes.
(XLSX)

S2 Table Survival analysis of main demographic, clinical and genomic variables. (XLSX)

S3 Table List of filtered and annotated somatic mutations (SNVS). (XLSX)
