# Supplementary material for: Genomic analysis of head and neck cancer cases from two high incidence regions
Source: PLoS One. 2018 Jan 29;13(1):e0191701. doi: 10.1371/journal.pone.0191701 (PMC5788352; doi:10.1371/journal.pone.0191701)

S1 Fig. Mutation calling validation. (A) Venn diagram of number of *TP53* mutations detected in the Gencapo Series. Example: *TP53*Asn239Asp mutation previously detected by Sanger sequencing (B) Plots of mutational calling showing an example of independent libraries sequenced from the same case

(A)

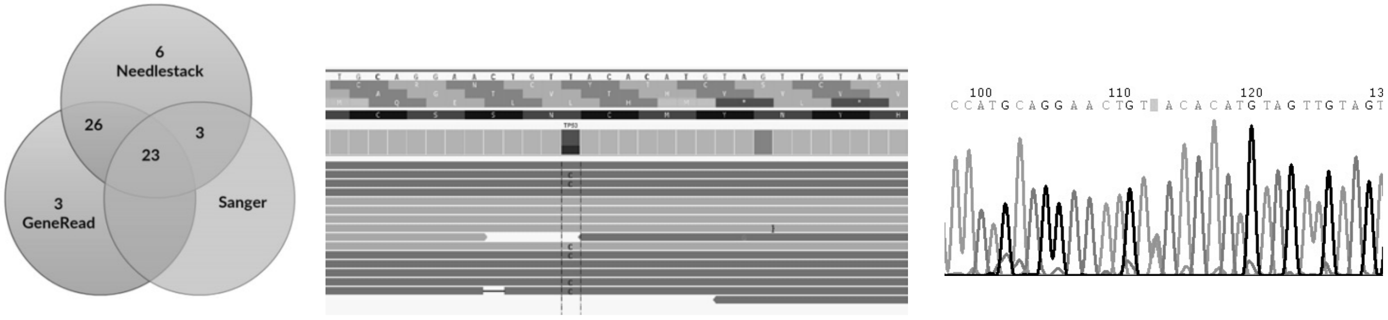

TP53 g.7577566T>C p.Asn239Asp

(B) *PTEN*:g.89692790G>T p.Asp92Tyr

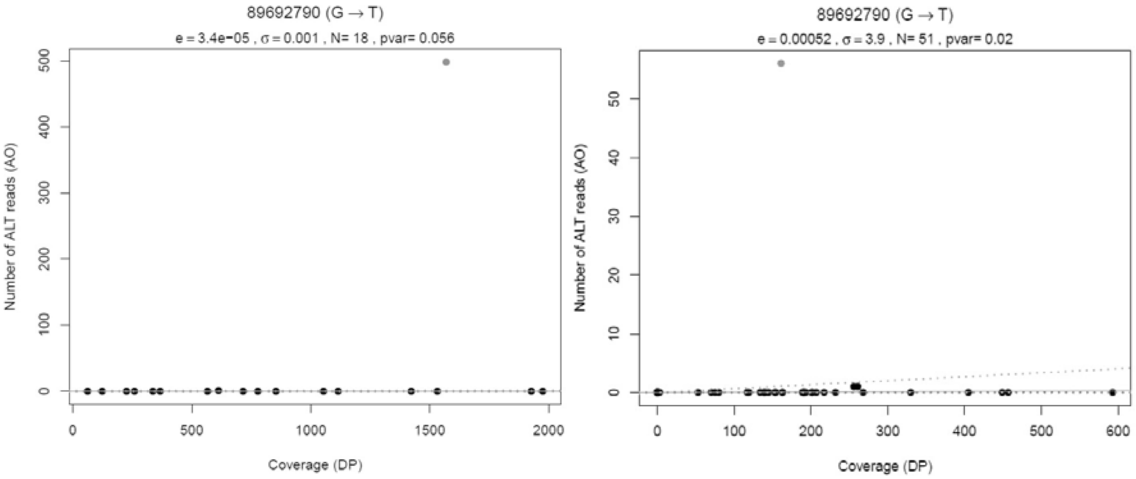

Supplement: S1 Fig — (A) Venn diagram of number of TP53 mutations detected in the Gencapo Series. Example: TP53Asn239Asp mutation previously detected by Sanger sequencing (B) Plots of mutational calling showing an example of independent libraries sequenced from the same case. (PDF) [file pone.0191701.s001.pdf]
