# Supplementary material for: Genomic analysis of head and neck cancer cases from two high incidence regions
Source: PLoS One. 2018 Jan 29;13(1):e0191701. doi: 10.1371/journal.pone.0191701 (PMC5788352; doi:10.1371/journal.pone.0191701)
Supplement: S2 Fig — (A) Mutually exclusive alterations between the 14 genes sequenced (Significance p-value of mutual exclusivity derived from the Z-score) (B) Co-occurrence of alterations (Significance p-value of co-occurrence derived from the Z-Score). Z score based on deviation of the observed mutations compared to expected, obtained by permuting events. (PDF) [file pone.0191701.s002.pdf]

S2 Fig. (A) Mutually exclusive alterations between the 14 genes sequenced (Significance p-value of mutual exclusivity derived from the Z-score) (B) Co-occurrence of alterations (Significance p-value of co-occurrence derived from the Z-Score). Z score based on deviation of the observed mutations compared to expected, obtained by permuting events.

(A)

	TP53	*NOTCH1*	*CDKN2A*	*CASP8*	*HRAS*	*PTEN*	*MLL2*	*PIK3CA*	*TP63*	*FAT1*	*RB1*	*IRF6*	*NSD1*	*FBXW7*
TP53														
NOTCH1	0,063													
CDKN2A	0,21	0,0097												
CASP8	0,46	0,59	0,77											
HRAS	0,0087	1	0,21	0,39										
PTEN	0,94	0,078	0,83	0,51	0,26									
MLL2	0,0082	0,12	0,69	0,86	0,67	0,076								
PIK3CA	2,80E-06	0,029	0,29	0,39	0,53	0,044	0,74							
TP63	0,0063	0,003	0,019	0,37	0,018	0,076	0,2	1,00E+00						
FAT1	0,15	0,26	0,26	0,21	0,6	0,04	0,94	0,25	0,013					
RB1	0,019	0,044	0,55	0,28	0,78	0,7	0,96	0,63	0,12	0,64				
IRF6	0,0058	0,44	0,72	0,45	0,82	0,84	0,98	0,96	0,049	0,91	0,95			
NSD1	0,23	0,72	0,21	0,48	0,45	0,12	0,56	0,036	0,085	0,12	0,22	0,76		
FBXW7	0,51	0,23	0,43	0,17	0,3	0,16	0,84	0,45	0,22	0,59	0,71	0,99	0,96	

(B)

	TP53	*NOTCH1*	*CDKN2A*	*CASP8*	*HRAS*	*PTEN*	*MLL2*	*PIK3CA*	*TP63*	*FAT1*	*RB1*	*IRF6*	*NSD1*	*FBXW7*
TP53														
NOTCH1	0,94													
CDKN2A	0,79	0,99												
CASP8	0,54	0,41	0,23											
HRAS	0,99	3,20E-05	0,79	0,61										
PTEN	0,059	0,92	0,17	0,49	0,74									
MLL2	0,99	0,88	0,31	0,14	0,33	0,92								
PIK3CA	1	0,97	0,71	0,61	0,47	0,96	0,26							
TP63	0,99	1	0,98	0,63	0,98	0,92	8	1,50E-12						
FAT1	0,85	0,74	0,74	0,79	0,4	0,96	0,059	0,75	0,99					
RB1	0,98	0,96	0,45	0,72	0,22	0,3	0,042	0,37	0,88	0,36				
IRF6	0,99	0,56	0,28	0,55	0,18	0,16	0,024	0,041	0,95	0,089	0,052			
NSD1	0,77	0,28	0,79	0,52	0,55	0,88	0,44	0,96	0,21	0,88	0,78	0,24		
FBXW7	0,49	0,77	0,57	0,83	0,7	0,84	0,16	0,55	0,78	0,41	0,29	0,012	0,039	
