# Supplementary material for: Genomic analysis of head and neck cancer cases from two high incidence regions
Source: PLoS One. 2018 Jan 29;13(1):e0191701. doi: 10.1371/journal.pone.0191701 (PMC5788352; doi:10.1371/journal.pone.0191701)

S3 Fig. Diagrams of mutation distribution in genes with frequent SNVs. Mutation colours represent: Green: Missense Mutations; red: Truncating Mutations (Nonsense, Nonstop, Frameshift deletion, Frameshift insertion, Splice site), black: Inframe Mutations (Inframe deletion, Inframe insertion). Circles colored with purple indicate residues that are affected by different mutation types at the same proportion.

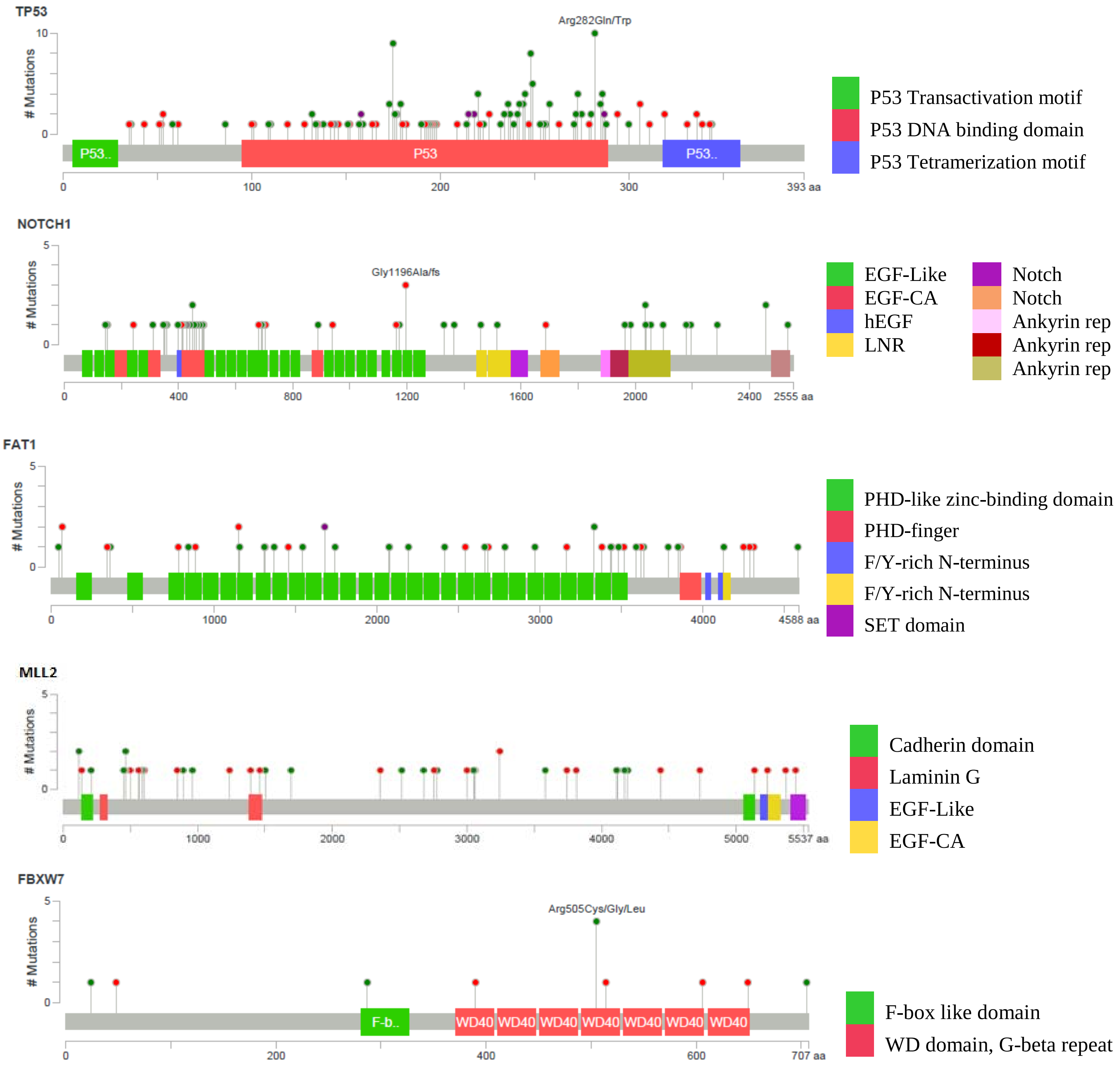

Supplement: S3 Fig — Mutation colours represent: Green: Missense Mutations; red: Truncating Mutations (Nonsense, Nonstop, Frameshift deletion, Frameshift insertion, Splice site), black: Inframe Mutations (Inframe deletion, Inframe insertion). Circles colored with purple indicate residues that are affected by different mutation types at the same proportion. (PDF) [file pone.0191701.s003.pdf]
