# Supplementary material for: Genomic analysis of head and neck cancer cases from two high incidence regions
Source: PLoS One. 2018 Jan 29;13(1):e0191701. doi: 10.1371/journal.pone.0191701 (PMC5788352; doi:10.1371/journal.pone.0191701)

S4 Fig. Mutational profile and copy number losses in HPV positive cases. (A) Mutational frequencies of the 14 genes sequenced in 15 HPV16E6 positive cases. (B) Comparison of Significant Focal copy number losses between HPV positive and HPV negative cases. (*) Regions significantly associated with overall survival

(A)

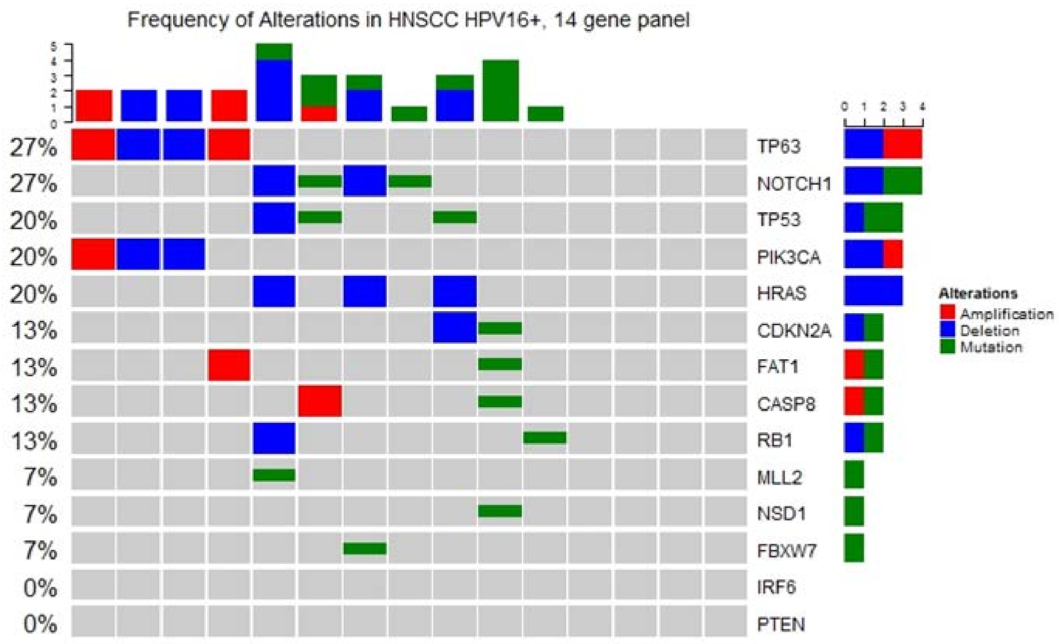

(B)

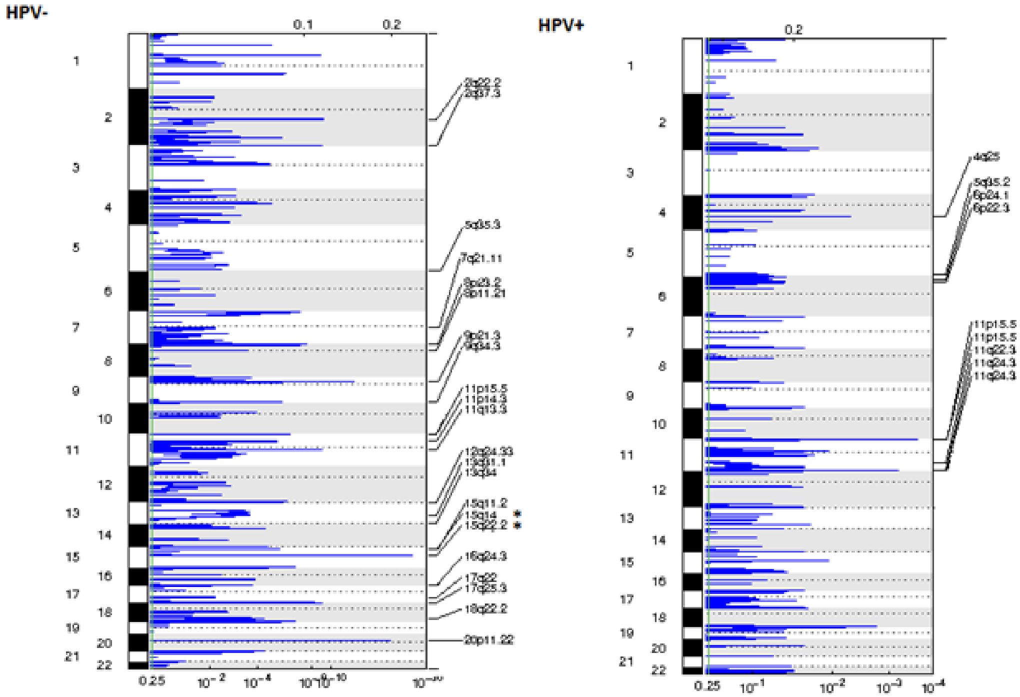

Supplement: S4 Fig — (A) Mutational frequencies of the 14 genes sequenced in 15 HPV16E6 positive cases. (B) Comparison of Significant Focal copy number losses between HPV positive and HPV negative cases. (*) Regions significantly associated with overall survival. (PDF) [file pone.0191701.s004.pdf]
